# Variation in the Visual Habitat May Mediate the Maintenance of Color Polymorphism in a Poeciliid Fish

**DOI:** 10.1371/journal.pone.0101497

**Published:** 2014-07-02

**Authors:** Jorge L. Hurtado-Gonzales, Ellis R. Loew, J. Albert C. Uy

**Affiliations:** 1 Department of Biology, Syracuse University, Syracuse, New York, United States of America; 2 Department of Biomedical Sciences, Cornell University, Ithaca, New York, United States of America; 3 Department of Biology, University of Miami, Coral Gables, Florida, United States of America; Arizona State University, United States of America

## Abstract

The conspicuousness of animal signals is influenced by their contrast against the background. As such, signal conspicuousness will tend to vary in nature because habitats are composed of a mosaic of backgrounds. Variation in attractiveness could result in variation in conspecific mate choice and risk of predation, which, in turn, may create opportunities for balancing selection to maintain distinct polymorphisms. We quantified male coloration, the absorbance spectrum of visual pigments and the photic environment of *Poecilia parae,* a fish species with five distinct male color morphs: a drab (i.e., grey), a striped, and three colorful (i.e., blue, red and yellow) morphs. Then, using physiological models, we assessed how male color patterns can be perceived in their natural visual habitats by conspecific females and a common cichlid predator, *Aequidens tetramerus*. Our estimates of chromatic and luminance contrasts suggest that the three most colorful morphs were consistently the most conspicuous across all habitats. However, variation in the visual background resulted in variation in which morph was the most conspicuous to females at each locality. Likewise, the most colorful morphs were the most conspicuous morphs to cichlid predators. If females are able to discriminate between conspicuous prospective mates and those preferred males are also more vulnerable to predation, variable visual habitats could influence the direction and strength of natural and sexual selection, thereby allowing for the persistence of color polymorphisms in natural environments.

## Introduction

The expression of exaggerated male traits, such as colorful ornaments or elaborate songs, often evolves under conflicting selective pressures [1], [2], [3]. Females may favor males with highly elaborate traits, but predators and other natural enemies may likewise prefer to target these attractive males [4]. Under such scenarios, the tradeoff in attractiveness to females and susceptibility to predators can favor the evolution of reduced conspicuousness [2] or alternative ways of communicating only detectable to conspecifics [5], [6].

The perception of elaborate signals depends on the physical properties of the habitat (e.g., ambient light and background, transmission spectrum of the medium) and the sensory parameters of the receivers assessing the signals [7]. Therefore, variation in any of these two components of the signaling environment may influence the trade-off between attractiveness and susceptibility to predation, and favor the evolution of alternative signal design (e.g., color, song frequency) that correspond to the variable environment [2], [8], [9]. Several studies offer support for this hypothesis by showing that variation in the photic environment (manakins [10], [11], African cichlids [12], [13], [14], bluefin killifish [15], anoles lizards [16], pentamorphic Sulawesi fish [17]) and visual physiology (African cichlids [18], sticklebacks: [19], bluefin killifish [20], [21], guppy [22], passerine and avian predators [23], crab spider [24]) can favor the transmission of specific signals that can be used by conspecifics and/or predators, and therefore promote the evolution and maintenance of color polymorphisms (see also [25]).

Although there is an established association between the photic environment and sensory physiology (e.g., [18], [19], [20], [26], [27]), only few studies have examined the simultaneous effects of these two factors on the outcome of female choice and male competition ([17], [19], [29], [30]; see also [9]). These studies suggest that variable sexual selection is an important selective force that can lead to the maintenance of male color polymorphisms. However, studies in which sexual selection may act in conjunction with natural selection via predation to maintain multiple color phenotypes are scarce (but see [31], [32], [33]). Here, we explore whether variable visual backgrounds and sensory physiology can directly affect the discrimination of distinct color phenotypes, as perceived by conspecific females and visual predators, and therefore maintain multiple color morphs in a single species.

**Figure 1:**
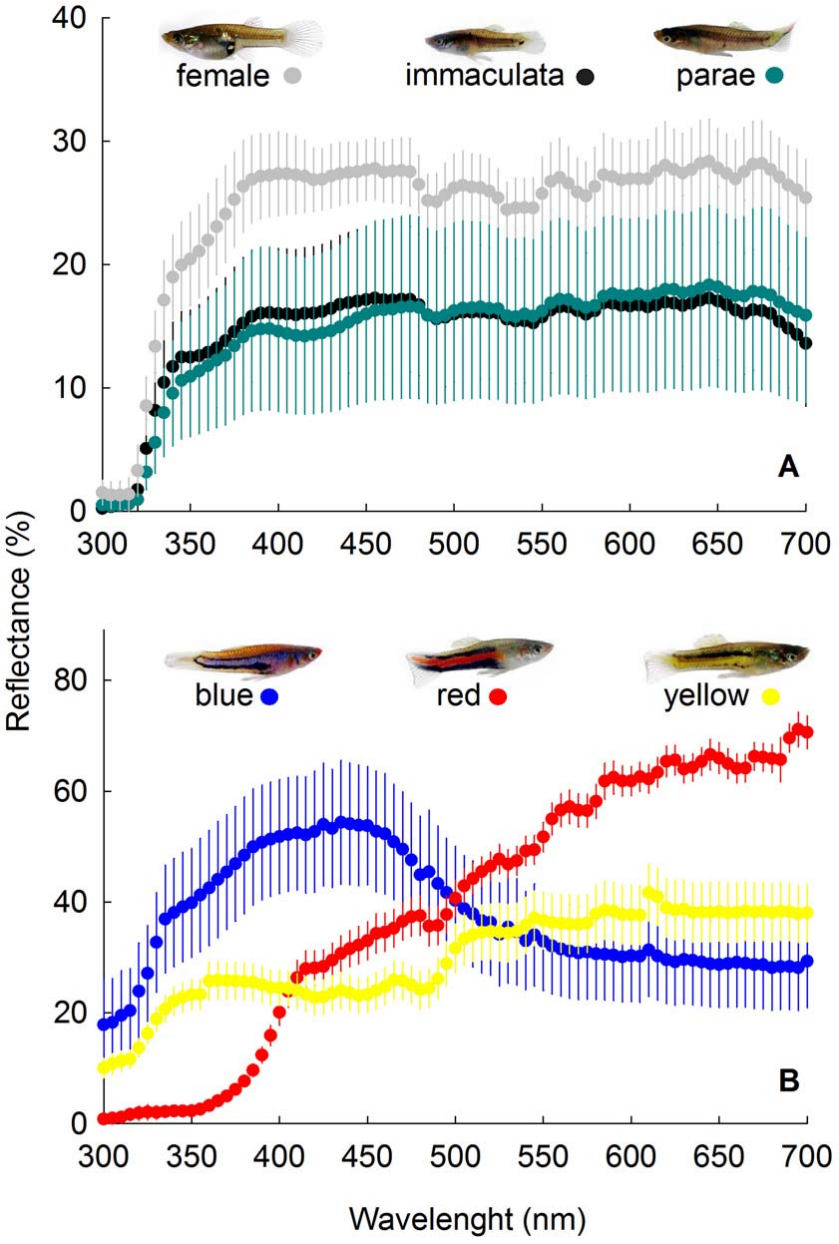
Mean (±1SE) reflectance of (A) female, and immaculata and parae males, and (B) blue, red and yellow males. Reflectance curves are from the means of 5 females and 5 males of each color morph.

Males of the poeciliid fish *Poecilia parae* exhibit five discrete Y-linked color morphs (Figure 1) [34]. These morphs include: (i) **immaculata**, the smallest and drab-colored males that resemble juvenile females, (ii) **parae**, the largest males that exhibit a striped tail and black vertical body bars that intensify during social interactions, and the (iii) **blue**, (iv) **red**, and (v) **yellow** males with colorful body flanks and intermediate body size [34], [35], [36]. Drab immaculata males use a sneaker strategy to gain copulations with females [37]. The other four male color morphs, in contrast, perform sigmoid courtship displays to advertise their color patterns to attract females for mating. During these sigmoid displays, males position their bodies to the front and sides of the receptive females with their dorsal and caudal fins fully extended [35]. Although the exposure of colorful sexual signals through elaborate courtships serves to attract females, such displays may also attract the attention of potential predators and thus may be costly in terms of increasing individual risk of mortality.

Field and laboratory studies suggest that the male color polymorphism in *P. parae* is stable across time [34] and may be maintained by a complex balance between the opposing effects of sexual and natural selection [36]. Laboratory experiments that controlled for male-male competition and standardized the photic environment (i.e., Sun-Glo linear fluorescent bulbs, Hagen, MA; ∼25 µE s^−1 ^m^−2^ mimicking natural, clear day light spectrum) revealed repeatable and variable female mating preferences for the colorful red, yellow and blue morphs. That is, most test females strongly preferred either red or yellow males but a few showed consistent preferences for blue males [36]. Likewise, *Aequidens tetramerus*, a common visual cichlid predator of *P. parae*, exhibited a visual bias for red and yellow males, suggesting that selective predation may offset the mating advantages of red and yellow males thus keeping them relatively rare in natural populations [36]. The large parae morph excludes sexual competitors through agonistic behaviors that are directed preferentially towards red, yellow and blue males, and rarely at drab immaculata males [38]. Exclusion of competitors resulted in increased mating success for parae males, indicating that these males use aggression as an alternative tactic to enhance their mating success. In contrast, the drab immaculata morph, is least preferred by females but can circumvent both female mate choice and male-male competition by mimicking juvenile females to deceive competitors and sneak copulations [36], [37]. Finally, blue males are able to gain mating opportunities by winning some aggressive interactions and by attracting some females [36], [38]. However, these small advantages may not fully explain the persistence of blue males at higher frequencies than red and yellow males in natural populations (see Figure 2 in [36]). Here, we test whether the heterogeneity of the visual environment impacts the conspicuousness of color signals by altering the perception of male color morphs by conspecifics and predators. Variation in perceived conspicuousness, in turn, may lead to variation in female choice and risk of predation. As such, we predict that observed variation in the visual background will result in variation in which color patches would be most conspicuous to females and cichlid predators. Such countervailing selection can promote the maintenance of striking and multiple male color morphs in natural populations.

**Figure 2:**
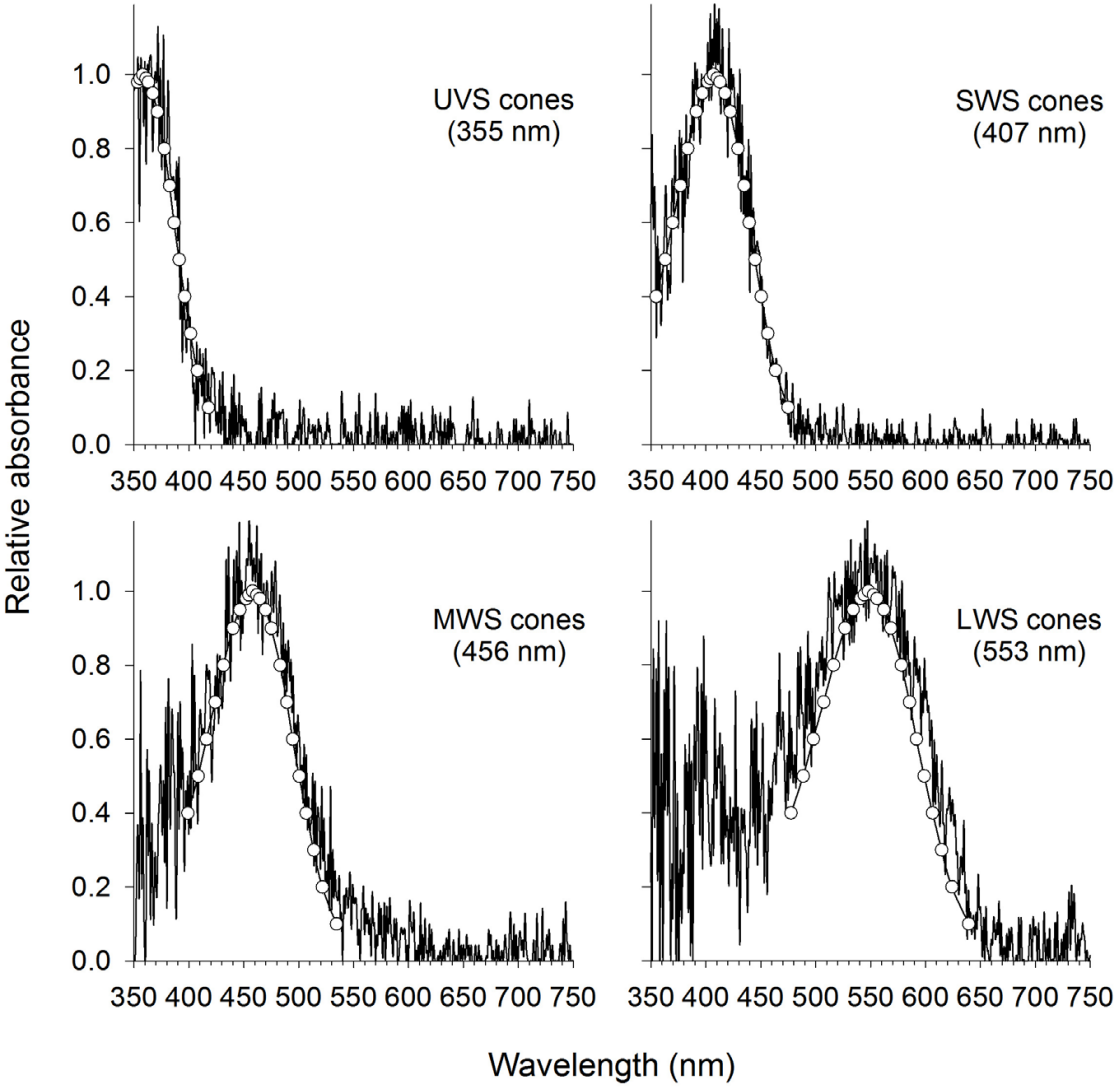
Example of the absorbance spectra for visual pigments of the cone photoreceptor cells of *Poecilia parae*. The best fitting templates (open circles) used to estimate the λ_max_ values of the pigments are shown overlaying the absorption curves.

## Materials and Methods

### Ethics Statement

The research was conducted under the Syracuse University Institutional Animal Care and Use Committee (IACUC) protocol number 06-014. Permit for field studies and collection of live individuals was issued by the Environmental Protection Agency, Republic of Guyana (Ref. 111207 BR 086). The present study only included the use of *Poecilia parae*, one of the most common poeciliids inhabiting freshwater coastal areas and did not involve any endangered or protected species.

### Study Sites

Study populations were located on the east (6° 47.2′ N, 58° 09′ W) and west (6° 41′ N, 58° 12′ W) sides of the Demerara River, Republic of Guyana (see [36] for characterization of sampling sites). We used the terms “east” and “west” populations to refer to two geographic locations where *P. parae* is found. Although both populations are contiguous, the east and west populations are separated by the Demerara River (ca. 1851 m wide), the third largest river in the Republic of Guyana that likely reduces gene flow between individuals of *P. parae* inhabiting the east and west populations. In January 2006, we established 15 permanent sampling sites (i.e., segments of the stream or drainages) at each population. Sampling sites were *ca*. 100–150 m apart from each other. From January to August 2006, we recorded the relative abundance of the five *P. parae* male color morphs, and that of their common predator, *Aequidens tetramerus* at 15 sites in the east and 15 sites in the west study populations. In January 2010, with the exception of individuals used for reflectance measurements (see below), males and females of *P. parae* were collected from the sampling sites and were transported to our laboratory at Syracuse University. Fish were maintained in 20 gal aquaria with treated water at 27±1°C, on a 12∶12 h light: dark cycle, and fed daily with live brine shrimp and Tetra-Min (Melle, Germany) flakes two times per day until used for microspectrophotometry analysis.

### Reflectance Measurements

From our 2010 field collection and immediately after capture, individuals of *P. parae* were sorted by sex and morph type, and housed in separate aquaria. Spectral reflectance of five randomly drawn females and five males of each morph were measured within 4 h of capture, allowing fish to acclimate and recover from stress due to handling/capture. Each specimen was individually anesthetized in an ice bath (∼2–3°C). The anaesthetized fish was then placed on its right flank on ice separated by a wet cloth, and its color patches on the flanks were measured using a portable spectrophotometer. We focused on color patches that are conspicuous, used during courtships and preferred by females (e.g., [34], [35], [36]). We excluded the measurements of color patches on the caudal and dorsal fins from our analyses because of the difficulties in gathering repeatable spectrometry readings of these small and translucent patches.

The reflectance of color patches was illuminated by a pulsed Xenon flash source (Ocean Optics PX-2; as in [10]) and measured with an Ocean Optics USB2000 spectrometer (Ocean Optics Inc., Dunedin, FL, USA) at a 45° angle to reduce specular glare. Reflectance scans were taken across 300–700 nm spectral range with the probe housed in a hollow, black anodized aluminum sheath with a 45° angled tip that contacted the fish’s skin. The anodized sheath insured that the Xenon flash was the only light source illuminating the color patch, that the distance between the probe and the color patch was constant at 0.5 cm, and that the angle of measure and size of the opening were constant at 45° and 1 mm diameter circle, respectively. To allow for comparison across scans, each reflectance scan was standardized with a spectrally flat 97% reflecting Spectralon white standard (Labsphere; North Sutton, NH, USA) and a dark standard.

### Microspectrophotometry

To characterize the spectral sensitivity of *P. parae* cones, seven adult males (immaculata: n = 2, parae: n = 2, blue: n = 2 and yellow: n = 1 morphs) and 10 adult females were used for microspectrophotometry (MSP) analyses at Cornell University, Ithaca, New York. Methods for MSP readings are described in detail in [39]. In brief, fish were housed in complete darkness for 24 hours. Then, individual fish was euthanized and the eyes enucleated under dim red light (Safelight N° 2, 15 W bulb, Kodak^©^, USA). All samples were prepared under infrared illumination (>800 nm, Safelight N° 11, Kodak^©^, USA) using image converters. The eyes were hemisected and the retinae removed while in buffer solution (cold phosphate buffer solution pH 7.4 supplemented with 6% sucrose). Retinae were then separated from the retinal pigment epithelium and macerated using razor blade fragments and tungsten needles [39]. A small sample of the retina was transferred to a cover slip and placed under a second cover slip edged with silicone grease. The 1.5 µm^2^ rectangular measuring aperture was produced by demagnification using a Leitz (Oberkochen, Germany) 180X quartz mirror objective. A Zeiss 100X Ultrafluar (0.85 NA) collected the transmitted light and focused it onto the photomultiplier photocathode. Retinal cells were selected individually. Determination of double cones was based on intact pairs of examined cells only. MSP scans were collected at 1 nm intervals scanning from 750 to 350 nm, and back from 350 to 750 nm [39]. Comparisons of both scans are commonly used to control for over-filtering and bleaching. The MSP settings and functioning used in this study is provided in detail in [40].

We used template fitting to determine the λ_max_ (the wavelength at maximum absorbance for a template-derived visual pigment best fitting the experimental data as defined by [20] and [39]). The process of determination of λ_max_ involved the following steps: (i) smooth the data, (ii) determine the peak absorbance (*X*_max_), (iii) normalize the absorbance curve, (iv) fit the templates, (v) calculate the standard deviation of λ_max_, and (vi) compare with the actual data and choose the best fit [39]. Then, pre-selected spectra were smoothed prior to normalization with the digital filter routine using Smooft [41]. For instance, a smoothed spectrum was overlaid on the raw data and visually compared for over-filtering or for spurious data points that had shifted the apparent maximum. If shifts were perceived, then the unsmoothed data were used. The peak absorbance (*X*_max_) used in the normalization represented the calculated maximum of the best fit Gaussian to the data points 20 nm either side of the estimated-by-eye absorbance maximum of the alpha band. Using *X*_max_, the data were then normalized using standard methods [42], [43]. Finally, normalized data were best-fitted using the A_1_ templates [44]. Since MSP wavelength error is ±1 nm, all visual pigments are reported to the nearest integer. Absorbance spectra from 30–40% of cells measured were retained for analysis since the information from other cells was of insufficient quality for template fitting. Hence we inspected more cells than the sample sizes we report in this study.

### Characterizing the photic environment: ambient light and visual background

We collected irradiance (i.e., ambient light) and radiance (i.e., background) scans at all sampling sites (east: n = 15 sites and west: n = 15 sites), where *P. parae* was common and all male color morphs present. Measurements were taken early in the morning (600–800 h) when male and female *P. parae* are most socially active [35]. We used a submersible Planar Irradiance collector (Hydro-Optics, Biology, and Instrumentation Laboratories, WA, USA) attached to an Ocean Optics USB2000 spectrometer (Ocean Optics Inc., Dunedin, FL, USA) to collect ambient light. At each site, irradiance was collected horizontally (i.e., downwelling) at a depth of 20 cm because *P. parae* inhabits shallow waters and most social and foraging activities occur within this depth [35].

Background radiance was measured by modifying the submersible irradiance probe with a black opaque cap of 40 mm in length and 4 mm in diameter to reduce the field of view (i.e., <5°) so that only light from a small solid angle can reach the detector surface. Radiance scans were taken every 15° angle until completing a semicircle, starting at an approximate location where an individual of *P. parae* was observed or likely to interact with females. The aim of these radiance scans was to measure the average visual background against which an individual would be perceived by a conspecific or predator. To allow for direct comparison among scans, the spectrophotometer fitted with the irradiance and radiance probes was calibrated with a standard light source (LiCor 1800-02; see [45]).

To characterize the spectral distribution of the ambient light and background for each sampling site in the east and west populations we calculated the spectral index λp50, the wavelength that halves the total number of photons between 300–700 nm [46]. The λp50 specifies a single value per habitat sampled in which the majority of photons are likely to be most concentrated. A high λp50 index suggests that the spectrum is more shifted to longer wavelengths. The λp50 index has been more recently used to determine the predominant spectral components characterizing different underwater photic regimes to examine fish visual signaling (e.g., [17], [28], [47]). We used one-way ANOVAs to test for differences in the estimated λp50 indices for the ambient light and visual backgrounds between sample sites at the east (n* = *15) and west populations (n = 15).

### Modeling the visual system of *Poecilia parae*

To quantify the chromatic Δ*S*) and luminance (*L*) contrasts of male color morphs as viewed through the eyes of *P. parae* conspecifics, we used the (i) reflectance spectra of a color patch (Figure 1), (ii) radiance spectra of the visual background, (iii) irradiance spectra of the ambient light illuminating the color patch, and (iv) spectral sensitivity of *P. parae.*

Our MSP data indicated that *P. parae* has seven types of cone photoreceptors located within single and double cones (see results section). Because it remains unclear if all seven of these cones are used in color or luminance discrimination, and if the double cones interact, we first modeled the visual system of *P. parae* as pentachromatic, assuming that double cones are neurally linked and able to operate as a single receptor channel (e.g., [47], [48], [49]). For this purpose, we considered the following combinations of photoreceptors: (i) 355–407–456–526–533, (ii) 355–407–456–526–543, (iii) 355–407–456–526–553; iv) 355–407–456–533–543, (v) 355–407–456–533–553. We then modeled a hexachromatic eye by including all photoreceptor types and assuming that individual members of double cones are used in color vision as independent spectral channels (e.g., [49]). Possible hexachromatic eyes would be composed of the following photoreceptors combinations (i) 355–407–456–526–533–543; and, (ii) 355–407–456–526–533–553. The choice of these two modeling approaches is based on our current knowledge of the differential expression of the SWS1, SWS2B, SWS2A, RH2 and LWS opsin subfamilies (often expressed in double cones) in Cyprinodontiformes, which suggests either a penta- or hexachromatic vision for these fishes (e.g., [50], [51], [52]).

As required by the Vorobyev-Osorio model, we also included (i) the relative frequencies of the cone classes and (ii) estimation of the Weber fraction for LWS cones in *P. parae*. The estimation of visual cone class densities were derived from published data on guppy retina based on the relative encounter rates of different cone classes inspected during retina preparations [53], [54]. In the absence of behavioral data on the visual thresholds of *P. parae*, the Weber fraction of the LWS cone was set at 0.05. This value was chosen as a conservative measure of visual performance, assuming that subjects can reliably detect a 5% change in stimulus intensity between objects or color patches (see other fish vision studies using a 5% Weber fraction: [55], [56], [57], [58]). All these data were processed using extended versions of receptor noise-limited color vision models for *P. parae* as developed by Morehouse *et al*. [33] and based on the Vorobyev-Osorio model [59]. Details of the extended models used to analyze penta- and hexachromatic visual systems are provided in appendix B of the electronic version of Morehouse et al. [33].

We evaluated the results of chromatic (Δ*S*) and luminance (*L*) contrasts of male color phenotypes as estimated by penta- and hexachromatic eye models using one-way ANOVAs. The pentachromatic model should represent a visual system with a limited subset of MSW/LWS photoreceptors compared to a hexachromatic model. Thus, we ask whether the exclusion of a MSW/LWS visual photoreceptor (i.e., turn a hexachromatic model to a pentachromatic model) will result in differences in color and luminance discrimination. We did not find any significant differences in color (all P>0.92) and luminance (all P>0.91) discrimination between pentachromatic and hexachromatic eye models. For simplicity, we therefore use the Δ*S* and *L* estimations based on hexachromatic eye for exploring the role of variable visual habitats in mediating variation in perceived conspicuousness of male signals (see below).

To test for perceived differences in conspicuousness of male color morphs within- and between populations by conspecifics, we used the 355–407–456–526–533–543 hexachromatic eye. We compared the color (Δ*S*) and luminance (*L*) contrast of the five morphs and females using nested ANOVAs. The independent variables included: population (east and west; n = 2), sites (sampling sites within-populations; n = 30 or 15 per population) and ‘morphs’ (male color morphs and female of *P. parae*; n = 6). The dependent variables were the color (Δ*S*) and luminance (*L*) contrasts estimated for each male color morph. ‘Population’ and ‘morph’ were considered as fixed factors given that we sampled two populations in Guyana, and that all male color morphs were present at all sampling sites. ‘Sampling sites’ were nested within ‘population’ and considered as a random factor. We used Fisher’s LSD pairwise tests to test for posthoc differences in the perceived conspicuousness or luminance of *P. parae* color morphs by conspecifics.

### Predator visual modeling

Cichlid fishes are common visual predators of colorful poeciliids and so oppose positive sexual selection (e.g., [2], [60]). Current evidence suggests that cichlids have trichromatic color vision and are capable of detecting short-wavelength light [31], [32], [61]. Hence, we specifically asked 1) whether the most conspicuous color morphs to *P. parae* are also conspicuous to a common predator, the cichlid *Aequidens tetramerus*, and 2) whether predators may play a role in mediating selection. Using the Vorobyev-Osorio trichromatic visual model [62], we quantified the color (Δ*S*) and luminance (*L*) contrasts of male color morphs against natural backgrounds as viewed through the eyes of a cichlild predator.

Because we do not have data on the spectral sensitivity of the cichlid *A. tetramerus* cones, we used published data from *A. pulcher*, a closely related, voracious predator of adult guppies in Trinidadian streams [31]. We used the following parameters for *A. pulcher* cone spectral sensitivity: SWS = 453 nm; MWS = 530 nm; LWS = 570 nm (as determined by [63]). The absorbance functions of *A. pulcher* were calculated using the Govardovskii’s pigment absorbance template equations [64]. For this purpose, we considered that the *A. pulcher* visual proteins are primarily composed of vitamin A2 (porphyropsin; [65]). The relative densities of photoreceptors used to estimate the perceptual threshold of the cichlid predator come from data collected for the South American cichlid, *Amphilophus longimanus*, a species that also inhabits clear freshwater streams [8], [66]. All the calculated perceived differences in conspicuousness of male color morphs within- and between populations by *A. tetramerus* were analyzed with nested ANOVAs, as detailed above.

### Predicting morph frequencies

As an indirect assessment of the effects of variable mate choice and predator susceptibility on the frequency of male color morphs, we constructed a backward, stepwise multiple regression model for each of the five morphs using morph frequencies as the dependent variable and the estimates of male conspicuous to conspecifics and predators as possible predictors. Morph frequency was estimated by dividing the total number of males counted for each morph by the total number of all males counted at each sampling site. As predictor variables, we used the color (ΔS) and luminance (*L*) contrast values of the particular male color morph at the sampling sites’ visual environments perceived by conspecifics or predators, as well as the relative abundance of predators. Morph frequency and predator abundance data were arcsine square root and log transformed, respectively. For each of the models, we inspected the variance inflation factors (VIF), which indicated little evidence of collinearity. All variables included in the model did not deviate significantly from normality (all p>0.46).

## Results

### Microspectrophotometry

The absorbance spectra of all the cone classes and individual rod cells from *Poecilia parae* [immaculata (n = 2), parae (n = 2), blue (n = 2), and yellow (n = 1) morphs, as well as 10 adult females] fit well with vitamin A_1_ pigment templates. Our MSP study revealed the presence of seven spectrally distinct types of cone photoreceptor arranged either as single or double cones. Most notably, the retina of *Poecilia parae* contains single cones with UV absorption at 355±1.4 nm (“UVS”; n = 11 individuals) and a violet/blue at 407±1.6 nm (“SWS”; n = 17 individuals). Values for λ_max_ of double cones ranged from 456 to 553 nm: 456±1 nm (n = 17 individuals), 526±0.8 nm (n = 17 individuals), 533±2 nm (n = 11 individuals), 543±1 nm (n = 17 individuals), and 553±1.9 nm (n = 5 individuals). The different types of double and twin cones were commonly found as 456/526 nm, 456/533 nm, 456/543 nm, 526/533 nm, 533/533, 543/543, and 553/553. Rod cells were also observed and had an estimated λ_max_ 503±1.2 nm (mean±SD, n = 17 individuals). Example MSP absorbance curves for these photoreceptor types are provided in Figure 2 and Figure S1.

### Characterizing the photic environment: ambient light and visual background

**Figure 3:**
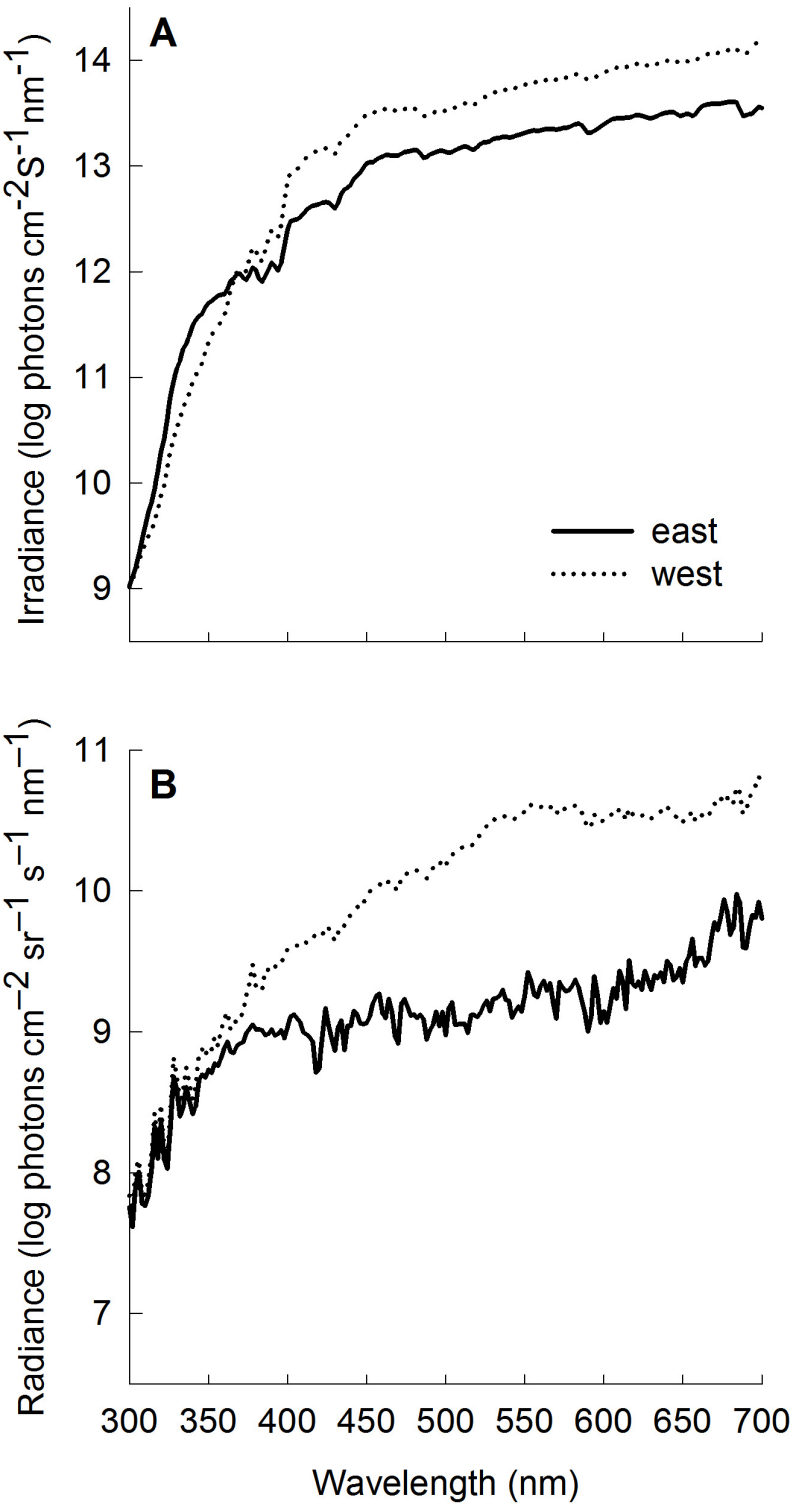
Mean (A) irradiance and (B) radiance spectra characterizing the sampling habitats in the east (solid line) and west (dotted line) populations where *P. parae* and their predators are found.

Unstandardized irradiance and radiance spectra of *P. parae* visual habitats are shown in Figure 3. The λp50 of the ambient light in the east (mean±s.e.: 568±3.64 nm) and west (576±3.64 nm) populations were not statistically different (ANOVA: *F*_1,28_ = 2.78, p = 0.11). However, the λp50 of the visual background (radiance) significantly differed between the east (545±2.68 nm) and west (562±4.45 nm) populations (ANOVA: *F*_1,28_ = 9.81, p<0.001), indicating more yellow shifted light in the west compared to the east population. The intensity of ambient light (east: 0.42±0.05, n = 15 sites and west: 0.72±0.14, n = 15 sites) and visual background (east: 0.18±0.03, n = 15 sites and west: 0.28±0.05, n = 15 sites) was consistently lower in the east population (Figure 3), which is consistent with the presence of more open habitats in the west compared to the east population.

### Modeling the visual system of *Poecilia parae*

**Figure 4:**
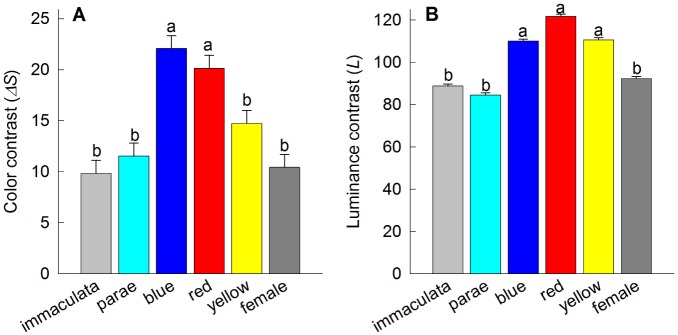
Mean (+1SE) (A) color contrast (Δ*S*) and (B) luminance contrast (*L*) as viewed through the hexachromatic visual system of *Poecilia parae*. Female and male color phenotypes with different letters are significantly different at P<0.01 (Fisher LSD post-hoc comparisons). Error bars represent 95% confidence intervals.

**Figure 5:**
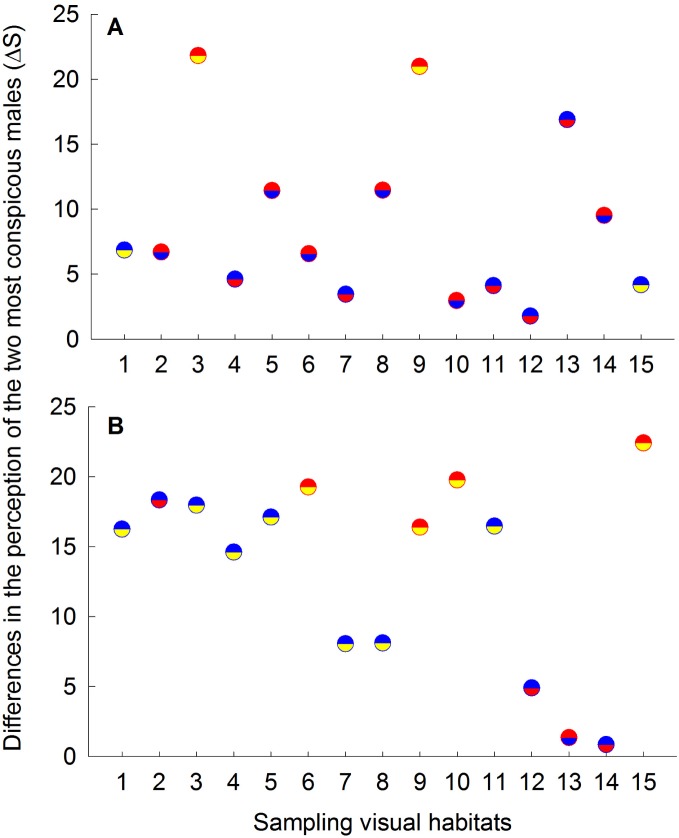
Differences in the perceived chromatic contrast between the two most conspicuous males as perceived by conspecifics across different sampling sites in the (A) east and (B) west populations. Differences were calculated by subtracting the Δ*S* values of the most conspicuous color morphs from the Δ*S* values of the second most conspicuous morph at each sampling site. Upper half of each circle represents the most conspicuous male color morph, while the lower half represents the second most conspicuous color morph at each site. Blue, red and yellow male color morphs are represented by blue, red and yellow colors, respectively.

When viewed through *P. parae* visual system, our models suggest significant differences in the perceived chromatic (F_5,145_ = 16.75, P<0.001; Figure 4a) and luminance (F_5,145_ = 41.62, P<0.001; Figure 4b) contrasts among the five male color morphs. These results were consistent between the pentachromatic and hexachromatic visual models. Specifically, on the average, blue and red males had the greatest color and luminance contrast values against their visual background, followed by yellow, parae and immaculata males (Figure 4a, b). Comparison of color contrast within (F_28,145_ = 28.18, P<0.00; Figure 5) populations (i.e., among sites) and between the east and west (F_1,145_ = 87.29, P<0.001) populations indicate significant variation in the conspicuousness of each male morph at each site, suggesting a strong effect of the ambient light and/or visual background. For instance, in the east population, blue males were more conspicuous than red males in localities 4, 7, 11, 12 and 13, while red males were more conspicuous than blue males in localities 2, 5, 6, 8, 10 and 14 (Figure 5a). No significant difference was found between the mean chromatic contrast and luminance contrast values of immaculata males (Δ*S*: 9.83±1.27; *L*: 88.78±1) and adult females (Δ*S*: 10.42±1.27; *L*: 92.35±1; Fisher LSD post-hoc P = 0.73 and P = 0.54; respectively), which is consistent with the hypothesis that immaculata males are perceived as females by conspecifics [37].

### Predator visual modeling

**Figure 6:**
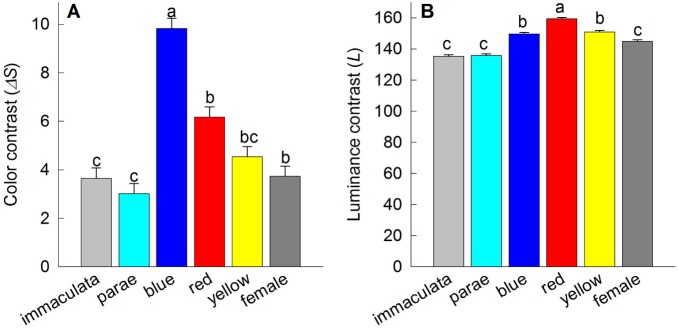
Mean (+1SE) (A) color contrast (Δ*S*) and (B) luminance contrast (*L*) as viewed through the trichromatic visual system of *Aequidens tetramerus*, a common cichlid predator. Female and male color phenotypes with different letter are significantly different at P<0.01 (Fisher LSD post-hoc comparisons). Error bars represent 95% confidence intervals.

**Figure 7:**
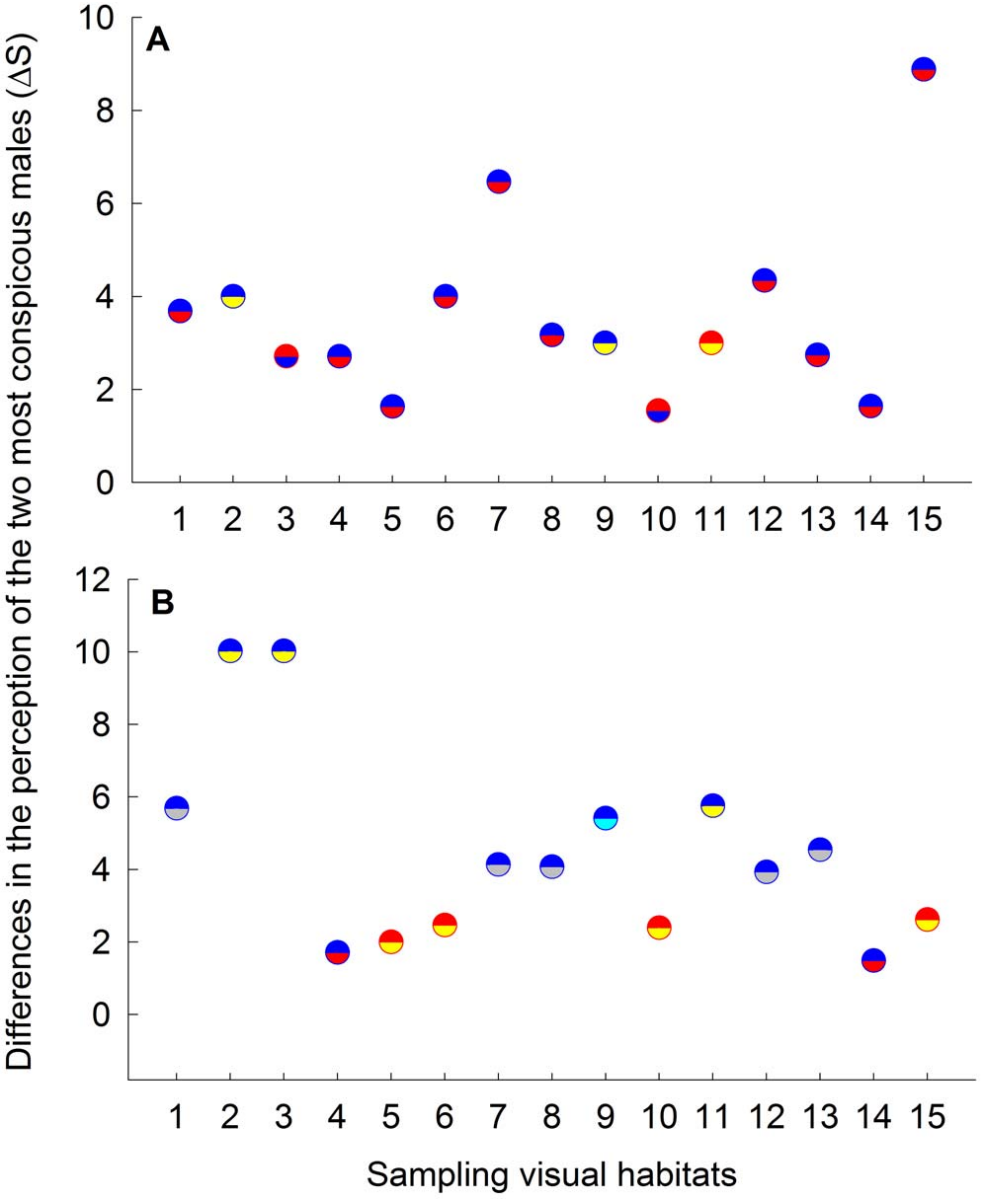
Differences in the perceived chromatic contrast between the two most conspicuous males as perceived by a common predator across different sampling sites in the (A) east and (B) west populations. Differences were calculated by subtracting the Δ*S* values of the most conspicuous color morphs from the Δ*S* values of the second most conspicuous morph at each sampling site. Upper half of each circle represents the most conspicuous male color morph, while the lower half represents the second most conspicuous color morph at each site. Blue, red, yellow, immaculata and parae male color morphs are represented by blue, red, yellow, gray and magenta colors, respectively.

Modeling a freshwater cichlid predator with a trichromatic color vision capable of detecting short-wavelength light during prey search, our results suggest that the male color morphs most preferred by *P. parae* females are also more conspicuous to cichlid predator, as estimated by perceived color (F_5,145_ = 35.92, P<0.001; Figure 6a) and luminance (F_5,145_ = 700.59, P<0.001; Figure 6b) contrasts. As in the modeling of *P. parae* perception, we found that the variable habitats also resulted in variation in the perception of males by cichlid predators within (F_28,145_ = 3.99, P<0.001) and between (F_1,145_ = 18.44, P<0.001) the east and west populations. Moreover, in ca. 45% of sampling sites in the east (i.e., sites 2, 5, 6, 8, 9, 11, 14; Figure 7a) and 20% of sampling sites in the west (i.e., sites 5, 9, and 13; Figure 7b) populations, we found that a male color morph that appear highly conspicuous to conspecifics under a particular background appeared less conspicuous to the cichlid predator. Considering the perception of immaculata and parae males, and females by a cichlid predator, they appear less conspicuous compared to other male color morphs against all natural visual habitats in terms of color contrast (Figure 6a) and luminance contrast (Figure 6b). However, our models suggest that the male color morphs of *P. parae* present lower color contrast but higher luminance contrast values to the visual predator (Figure 6a, b).

### Predicting morph frequencies

The factors that predicted morph frequency varied among color morphs (Table 1). For blue males, their chromatic conspicuousness (ΔS) to conspecifics emerged as the only significant predictor of the frequency of blue males (Table 1). That is, blue males were found in higher frequencies in areas where their color contrast is relatively high, explaining 21% of the variation in blue male frequency across sites. For red and yellow males, the relative abundance of predators emerged as a significant predictor of their frequencies across sampling sites (Table 1). Finally, the frequencies of the parae and immaculata morphs were predicted by their conspicuousness to cichlid predators (Table 1).

**Table 1:** Significant predictors of morph frequency across sampling sites in *Poecilia parae.*

Morphs	Significant predictors	R^2^	β'	F	P	df
1. immaculata	Color contrast (predator)	0.19	−0.03	6.53	0.02	1, 28
2. parae	*Whole model*	0.30		5.72	0.01	2, 27
	• Color contrast (predator)		0.04	8.43	0.01	1, 28
	• Luminance contrast (predator)		0.01	9.18	0.01	1, 28
3. blue	• Conspecific color contrast	0.21	0.01	7.40	0.01	1, 28
4. red	*Whole model*	0.34		6.83	0.00	2, 27
	• Luminance contrast (*P. parae*)		0.00	5.46	0.03	1, 28
	• Predator relative abundance		−0.10	12.43	0.00	1, 28
5. yellow	*Whole model*	0.36		4.40	0.01	3, 26
	• Luminance contrast (*P. parae*)		0.00	5.60	0.03	1, 28
	• Luminance contrast (predator)		−0.01	5.19	0.03	1, 28
	• Predator relative abundance		−0.08	4.45	0.05	1, 28

Note: Each model was constructed using backward stepwise multiple regression analysis with color and luminance contrasts as determined for conspecifics, color and luminance contrast as determined for predators, and the relative abundance of *Aequidens tetramerus* cichlid predator as independent variables. Standardized regression coefficients (β') and significance tests are shown for each significant predictor variable. Normality Test (Shapiro-Wilk) P>0.46.

## Discussion

Our results indicate that the perceived conspicuousness of the five *P. parae* male color morphs, as estimated by their color and luminance contrasts against the natural visual background and visual parameters of conspecifics, varied across sampling sites within populations. Such variation was primarily driven by the spectral properties of the variable visual background. In some localities, for instance, blue males were more conspicuous to conspecifics than the carotenoid-based red and yellow males (Table 1, Figure 5), which are the most attractive males under controlled laboratory conditions with full-spectrum light and a brown background [34], [36], [67]. If female mate choice is influenced by male conspicuousness, as shown in several fish species (e.g., [17], [68], [69]), variation in color and luminance conspicuousness of the different male morphs mediated by variable visual habitats may result in variable female mate choice. Variation in female mate choice, in turn, should allow for the persistence of distinct male color polymorphisms within populations (e.g., [9], [70], [71]). Consistent with this hypothesis, we found that the perceived color contrast of blue males by conspecifics positively predicted their relative abundance across sampling sites within populations (Table 1).

### Variation in sensory physiology

Our results suggest the presence of seven cone types in *P*. *parae*, representing the UV, SWS, MWS and LWS cone classes. We are confident that we have identified all the opsins expressed in recordable amounts in *P. parae* retinae. The MSP data further suggests that some individuals may be expressing at least one different LWS as a double cone with peak absorption at 526/533 nm or as a twin cone with λ_max_ of 553/553 nm but never both. At this point, we have no conclusive evidence suggesting that the LWS photopigments are either polymorphic or sex-linked (as in guppies [22], [52], [54], [72], [73], [74]), despite the extreme male color polymorphism. Our sample size is low and more in depth sampling across populations may perhaps reveal a sex-linked pattern of polymorphism.

At the molecular level, there is evidence indicating that *P. parae* expresses two LWS opsins (i.e. P180 and S180) found also in guppies and other poeciliids [75]. In guppies, for instance, the P180 LWS is expressed at a λ_max_ of 512 nm, whereas the S180 LWS may have a λ_max_ of 560 nm. From these two opsins only the latter appears to differ among populations [76]. At this stage, a combination of MSP, opsin gene sequences and spectral analysis of recombinant pigments among populations may help identifying cone complements and offer a better understanding of how visual sensitivities can influence the maintenance of color polymorphism in *P. parae*.

### Interaction between natural and sexual selection allows for the maintenance of 5 color morphs

Courtship traits are expected to evolve under conflicting selective forces. Sexual selection should favor the evolution of conspicuous traits, whereas natural selection via predation should agonistically work to decrease trait conspicuousness [2], [4]. In fact, several studies indicate that females and predators share a sensory bias for conspicuous signals (e.g., [2], [6], [23], [77]). Under such constraints, males should evolve less elaborate signals. Alternatively, signals can evolve ways to be more conspicuous to conspecifics than predators. For instance, at specific visual backgrounds, colorful plumage in songbirds can remain conspicuous to conspecifics while remaining inconspicuous to predators [23]. Likewise, ultraviolet signaling in northern swordtails (*Xiphophorus* spp) appear to work as a ‘private channel’ of communication between conspecifics, as predators do not see well in the UV spectrum [6]. Indeed, there is evidence that a voracious guppy cichlid predator (i.e., the Trinidadian pike: *Chrenicicla frenata*) lacks SWS1 opsin; therefore, this species may be relatively insensitive to the UV visual spectrum [61]. Thus, the existence and the role of a “private channel” in *Poecilia parae* remains an interesting open question to be resolved.

In *P. parae*, laboratory experiments of predator preferences show consistent bias for the carotenoid-based red and yellow males, which are also attractive to females (e.g., [36]). Our visual models confirm that indeed the red and yellow, as well as blue, color patches are more conspicuous to a piscivorous fish than the duller immaculata morph and females in their natural environment. These differences, however, depend on whether cichlid predators use either their color or luminance contrasts in detecting prey. The color contrast values indicates, for instance, that under some visual backgrounds a male color morph that is most conspicuous to conspecifics may be the least conspicuous to the predator. However, using luminance contrast, our results suggests that the colorful morphs appear very conspicuous to predators and thus may be preferentially targeted as prey. This hypothesis is supported by our results indicating that the relative abundances of yellow and red males are negatively predicted by their perceived luminance contrast to predators.

These differences in perceived conspicuousness appear to translate into differences in risk of predation mediated by variation in the background against which a particular male color morph is viewed. However, *P. parae* color patches were always more conspicuous to conspecifics and apparently less to predators, which may suggest that males reduce the costs of bearing colorful traits by displaying in visual conditions more conspicuous to conspecifics but less conspicuous to predators (as in [31]).

Combining our current results with our previous work [36], [37], [38], we can propose a more complete scenario of the mechanisms that interact to favor the persistence of the five distinct male color morphs in *P. parae.* First, both red and yellow males (i.e., carotenoid-based patches) are strongly preferred by females as potential mates under standardized laboratory conditions. Red and yellow males, however, are also preferentially targeted by visual predators under the same visual conditions [36]. Indeed, the results of the multiple regression models (Table 1) suggest that red and yellow morph frequencies are limited by the relative abundance of predators across different habitats. This tradeoff between natural and sexual selection could explain why red and yellow males are consistently rare throughout the years (e.g., [2]), and may provide opportunities for alternative mating strategies to invade. Second, drab immaculata males that resemble juvenile females are least attractive to females (e.g., [67]), and so they use a sneak copulation strategy to circumvent both female mate choice and male-male competition (e.g., [37]). In further support of this hypothesis, we found that immaculata males are similar to females in perceived color contrast, which likely enhances their ability to sneak copulations and avoid male-male aggression. The sneaker strategy of immaculata males is also enhanced by a relatively larger investment in testes, making them perhaps more competitive in sperm competition [37]. Third, the parae morph gain mating through elaborate courtships, and more significantly, by preventing other males from gaining access to females and/or modifying female choice after successful aggressive interactions with competitors [38]. That is, parae males specialize in agonistic interactions to enhance their mating success. Finally, our results in this study suggest that blue males, in certain visual conditions, are more conspicuous than all other morphs, including the highly-preferred red morph (Figure 5), and that perceived conspicuousness to conspecifics positively predicts blue male frequency (Table 1). These results suggest that conspicuousness may influence female mate choice (as in [68]) and thus the reproductive success of blue males (as in [17]). Variable visual conditions may therefore allow blue males to invade and persist in the population. These advantages, however, may be offset by predation, as blue males are also more conspicuous to predators, perhaps explaining why the three colorful males are found in lower abundances compared to the drab immaculata and less colorful parae morphs [36]. For instance, pike cichlid targets male guppies exhibiting large/numerous blue/iridescent spots as prey [2], and it has been currently established that pike cichlids are capable of detecting short-wavelength light [61]. All together, our results here and from previous work suggest that a complex interaction between natural and sexual selection allows for the remarkable persistence of five *P. parae* color morphs in nature.

## Conclusions

Recent studies provide support for the role of the visual background in generating and/or maintaining color polymorphisms by altering the direction of natural and sexual selection. In the guppy *Poecilia reticulate* the visual background varies with the lighting environment in natural streams, affecting the spatial and temporal operation of sexual selection. This, in turn, generates opportunities for the maintenance of quantitative variation in male coloration [68]. Likewise, in the pentamorphic Sulawesi fish *Telmatherina sarasinorum*, yellow and blue males are highly abundant in habitats that enhance their contrast from the visual background, which presumably augments their reproductive fitness [17]. In our study in *P. parae*, conspecifics perceive red males as the most conspicuous color morph, followed by blue then yellow males. However, variation in the visual habitat results in the blue morph being more conspicuous than red and yellow males, which in turn, predicts their relative abundances across sampling sites. Sexual selection favoring the most conspicuous males can therefore favor different color morphs under variable lighting conditions. That is, variation in the visual environment could lead to changes in the strength and direction of sexual selection. Our study therefore suggests an important role for environmental heterogeneity in favoring the maintenance of striking color polymorphisms.

## Supporting Information

Figure S1 **Example absorbance spectra for visual pigments of the rod photoreceptor cells of** ***Poecilia parae*** **.** The raw absorbance spectra, derived by MSP, are overlain with smooth curves calculated from the best-fitting A2-type chromophore visual pigment curve.
(DOCX)
