# Supplementary material for: Variation in the Visual Habitat May Mediate the Maintenance of Color Polymorphism in a Poeciliid Fish
Source: PLoS One. 2014 Jul 2;9(7):e101497. doi: 10.1371/journal.pone.0101497 (PMC4079317; doi:10.1371/journal.pone.0101497)

Figure S1. Example absorbance spectra for visual pigments of the rod photoreceptor cells of *Poecilia parae*. The raw absorbance spectra, derived by MSP, are overlain with smooth curves calculated from the best-fitting A2-type chromophore visual pigment curve.


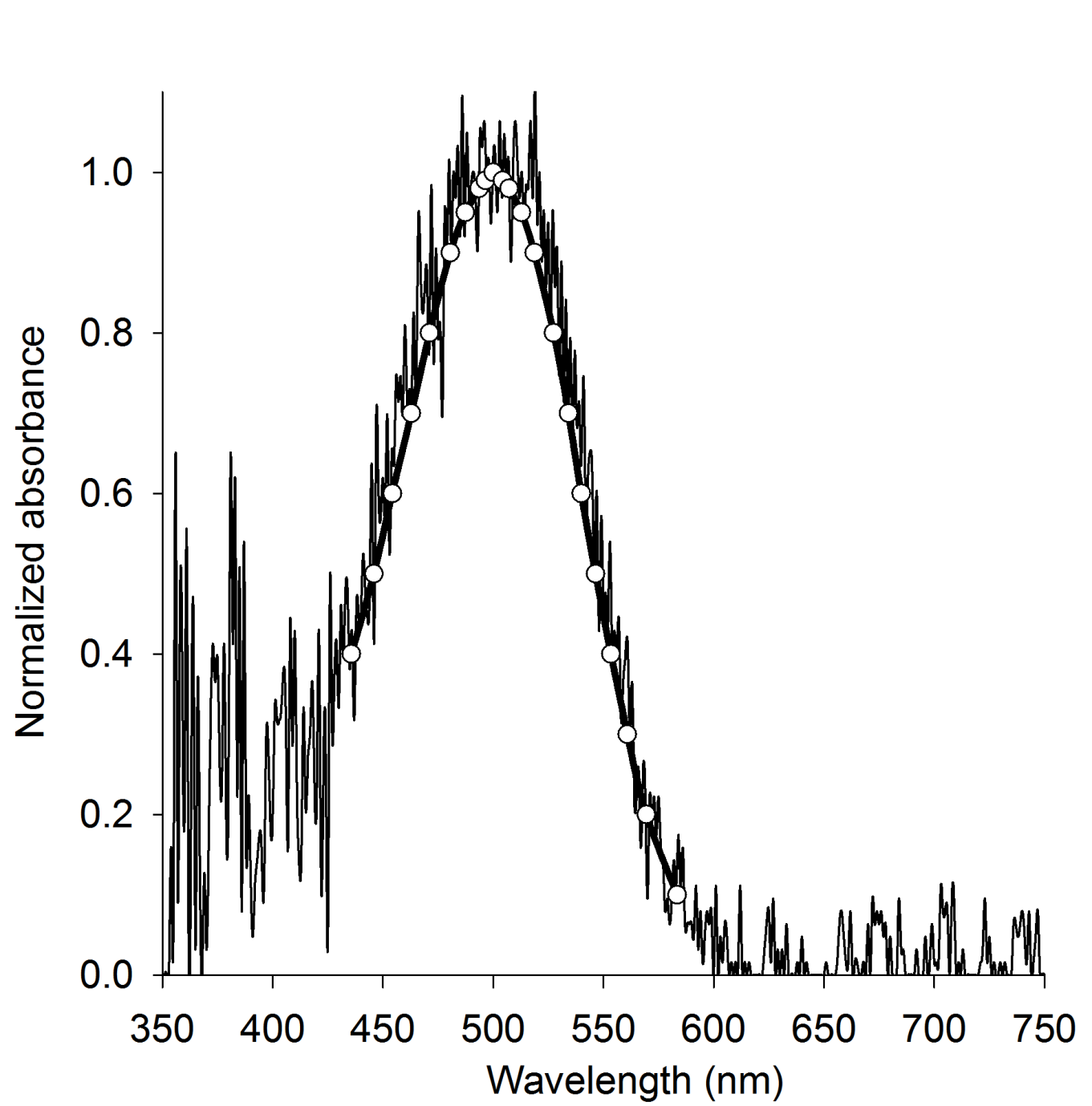

Supplement: Figure S1 — Example absorbance spectra for visual pigments of the rod photoreceptor cells of Poecilia parae . The raw absorbance spectra, derived by MSP, are overlain with smooth curves calculated from the best-fitting A2-type chromophore visual pigment curve. (DOCX) [file pone.0101497.s001.docx]
